# Slicing overcomes the bacterial cell wall barrier to fluorescence *in situ* hybridization

**DOI:** 10.1128/spectrum.02001-25

**Published:** 2025-12-10

**Authors:** Jennifer Gundrum, S. Tabita Ramirez-Puebla, Jessica L. Mark Welch, Gary G. Borisy

**Affiliations:** 1 ADA Forsyth Institute, Somerville, Massachusetts, USA; The Ohio State University College of Dentistry, Columbus, Ohio, USA

**Keywords:** sectioning, Gram-positive bacteria, confocal microscopy, fluorescence *in situ* hybridization, polymicrobial biofilm, cell wall, microbial ecology, imaging

## Abstract

Simultaneous visualization of all bacterial species in a polymicrobial biofilm by fluorescence *in situ* hybridization (FISH) remains a challenge because bacterial taxa respond differently to the hybridization procedure. This heterogeneity is due, in part, to the cell wall barrier of Gram-positive taxa. Enzymatic procedures required to permeabilize the cell walls of Gram-positive microbes can result in the disruption or loss of Gram-negative bacteria. Here, we demonstrate a procedure that enables the hybridization of difficult-to-hybridize bacteria while preserving the microarchitecture of all bacteria within a biofilm. The key feature of the procedure is to physically cut through the cell wall, thus allowing probe entry. We first embed the sample in a covalently crosslinked, glycol methacrylate resin to preserve the structure of the biofilm. Embedment is followed by sectioning and hybridization of material with the methacrylate still in place. We tested the procedure on common oral species, including difficult Gram-positive taxa, and found marked improvement in hybridization; both average signal intensity and homogeneity of hybridization were improved as compared to standard whole cell mount procedures. Our results confirm that the cell wall is the major barrier preventing efficient hybridization in whole mount samples. By physically overcoming the cell wall barrier, our protocol provides a universal procedure to visualize all bacteria in a polymicrobial community.

**IMPORTANCE:**

It has long been recognized that the major barrier to efficient *in situ* hybridization of bacteria is the cell wall, with Gram-positive bacteria generally being the most problematic. Because enzymatic methods that facilitate hybridization of Gram-positive bacteria can result in the loss of Gram-negative bacteria, visualization of both kinds of bacteria simultaneously is often not feasible. In this study, we use embedding and sectioning to establish a universal approach for the simultaneous visualization of all bacteria within a microbial community while preserving its microarchitecture. We show that the mechanism underlying the approach is the physical slicing of the bacterial cell, thus obviating the barrier posed by the cell wall. These findings will benefit researchers within the microbiology community interested in complex microbial communities.

## INTRODUCTION

One of the most effective approaches for imaging microbes at the single-cell level is fluorescence *in situ* hybridization (FISH) of oligonucleotide probes to complementary sequences in ribosomal RNA molecules. This approach, first reported 35 years ago (1, 2), has found multiple applications in basic science, microbial identification, phylogenetics, and medical diagnostics (3–8). Typically, microbes are first fixed as whole cells and then exposed to probes under suitable hybridization conditions. Such whole-cell hybridization requires that probes pass across the bacterial cell wall to access target nucleotide sequences inside the cell. Although whole-cell FISH has been generally successful with Gram-negative bacteria, Gram-positive bacteria have frequently proved problematic. The potential barrier to penetration presented by many Gram-positive bacteria was recognized very early in the development of bacterial *in situ* hybridization (3, 9). Success in hybridization of Gram-positive bacteria was accomplished through enzymatic procedures designed to digest proteoglycans of the cell wall (9–14), as well as by bacteriophage-encoded autolytic enzymes (15). These early results supported the conclusion that the cell wall was indeed a barrier to probe penetration. However, the enzymatic procedures required customization to individual taxa.

A major goal in microbiome research is the simultaneous visualization of all members of a microbial community. Significant progress toward achieving this goal has been made through careful design of probes and multiplexed or sequential FISH imaging (16–23). However, full success has been hindered by the typical complexity of microbial communities, which often include both Gram-positive and Gram-negative members. Permeabilization procedures that work efficiently with Gram-negative bacteria often fail with Gram-positive bacteria, giving heterogeneous hybridization or no hybridization at all. Conversely, the strong permeabilization procedures required for some Gram-positive bacteria result in disruption of cell integrity or loss of Gram-negative bacteria. With careful attention to conditions, multiplexed FISH imaging of an *in vitro* biofilm comprised of six Gram-positive and negative bacteria was accomplished (24). However, no single robust procedure has yet been found to enable simultaneous visualization of all bacteria in a complex microbial community.

FISH labeling of bacteria in sections has been reported for a variety of tissue sample types, including oral (17, 25–27), gut (28–30), skin (31), endocarditis (32, 33), colon cancer (34), and kelp (35). However, in nearly all these studies, sectioning was employed out of necessity because the samples were tissues as opposed to dispersed cells or biofilms. We have applied embedding and sectioning to *ex vivo* oral biofilms (17, 36) to obviate some of the difficulties in imaging thick biofilm samples by whole mount procedures optimized for dispersed cells. Our results suggested that sectioning could improve visualization as compared to whole mount preparations. However, none of these studies specifically investigated whether sectioning *per se* could mitigate the barrier posed by the walls of Gram-positive bacteria.

Here, we report a simple procedure that enables labeling of both Gram-negative and Gram-positive bacteria. It is based on the time-honored method of embedding and sectioning, which has been a foundational part of cell biology for over a hundred years (37). Instead of chemical or enzymatic treatments that need to be customized for individual bacterial species, sectioning is a mechanical procedure. Bacteria are embedded in a plastic resin, which is then sliced into thin sections by a sharp knife using a microtome. The effect is to cut the cell wall, enabling entry of probes directly into the cell, thus obviating the cell wall barrier. We demonstrate and quantify the efficacy of embedding and sectioning for hybridization of problematic bacteria, and we show that the mechanism of improved hybridization results from physically slicing the cell open.

## RESULTS

### Physical sectioning improves hybridization

We evaluated whether embedding and sectioning improve hybridization in bacterial cells by comparing the standard procedure of hybridizing oligonucleotide probes to whole bacterial cells with a procedure in which the cells are first embedded in a plastic resin, sectioned, and then hybridized.

To make the comparison as equivalent as possible, all steps in the procedure other than whole cell vs section hybridization were kept the same. Bacteria for both procedures were prepared from the same cultures; following cell washing and fixation, one part was stored as whole cells, and one part was pelleted, embedded in resin, and sectioned. Whole cells and sections were subjected to hybridization with the same oligonucleotide probes under the same stringency and washing conditions. Raw confocal microscopy images were acquired with the same excitation, optical, and detector settings, and final processed images were prepared after identical linear adjustment to optimize display. Thus, intensities of hybridization as seen in the presented images are directly comparable.

**Fig 1:**
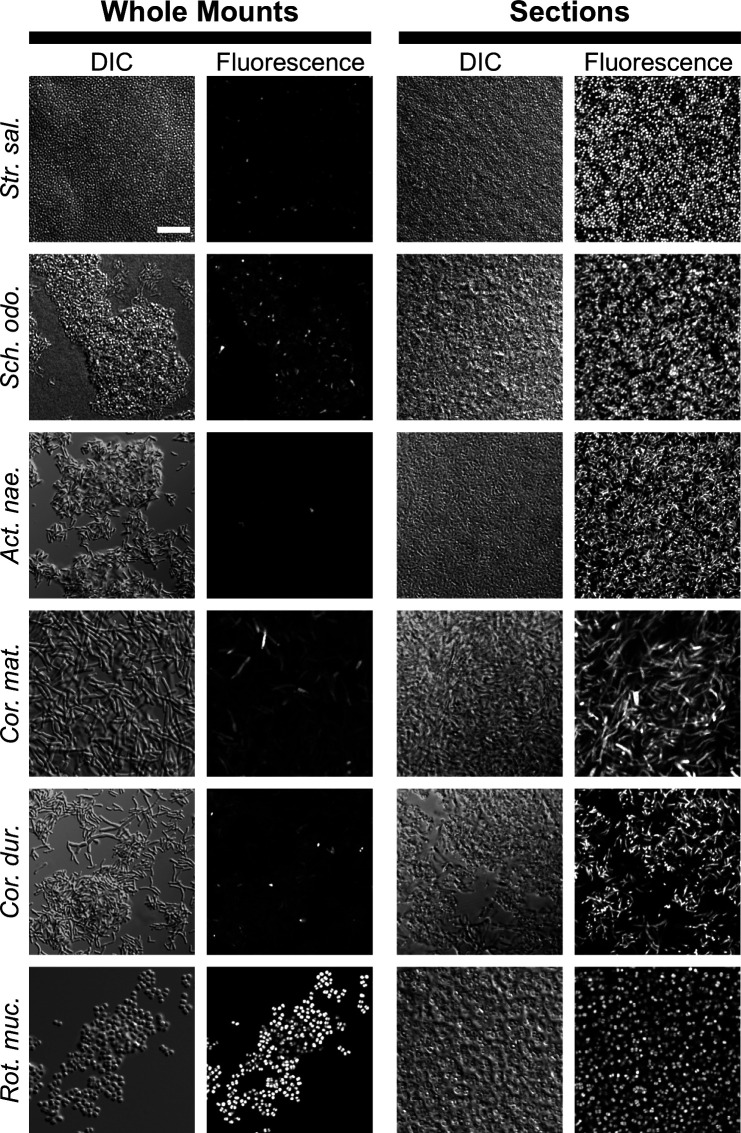
Physical sectioning improves hybridization of Gram-positive bacteria. Bacterial cells were hybridized under identical conditions either as whole cell mounts (left) or after embedding and sectioning (right) and imaged with confocal microscopy. Differential interference contrast (DIC) microscopy images are shown to display all cells in the field of view. Fluorescence images display hybridization signal from the FISH probe Eub338-Dy505. Across individual species, image acquisition and display settings were kept constant to allow comparison between the two sample preparations. In whole mounts, only a small fraction of the cells stain brightly. In sections, fluorescence signal is homogeneous, and most cells stain brightly. *R. mucilaginosa*, which hybridizes well in whole cell mounts, is shown as a positive control. Scale bar = 10 microns.

We focused our comparison on Gram-positive oral bacteria that are known to be difficult to hybridize. These include members of the *Actinomyces*, *Corynebacterium*, *Schaalia*, and *Streptococcus* genera. The results (Fig. 1) were striking. For each of the problematic Gram-positive bacteria, *Streptococcus salivarius, Schaalia odontolytica, Actinomyces naeslundii, Corynebacterium matruchotii,* and *Corynebacterium durum*, whole-cell hybridization resulted in heterogeneous hybridization with only a small fraction of the cells staining brightly. In contrast, the embedding and sectioning approach resulted in relatively homogeneous hybridization with most cells staining brightly. Some Gram-positive bacteria, such as *Rothia mucilaginosa,* hybridize well in whole cell mounts. We tested whether embedding and sectioning would offer any stronger hybridization in such a case. In contrast to the difficult Gram-positive bacteria, *R. mucilaginosa* exhibited a similar homogeneous signal under both conditions, suggesting that the cell wall for this taxon did not pose a significant barrier to hybridization.

**Fig 2:**
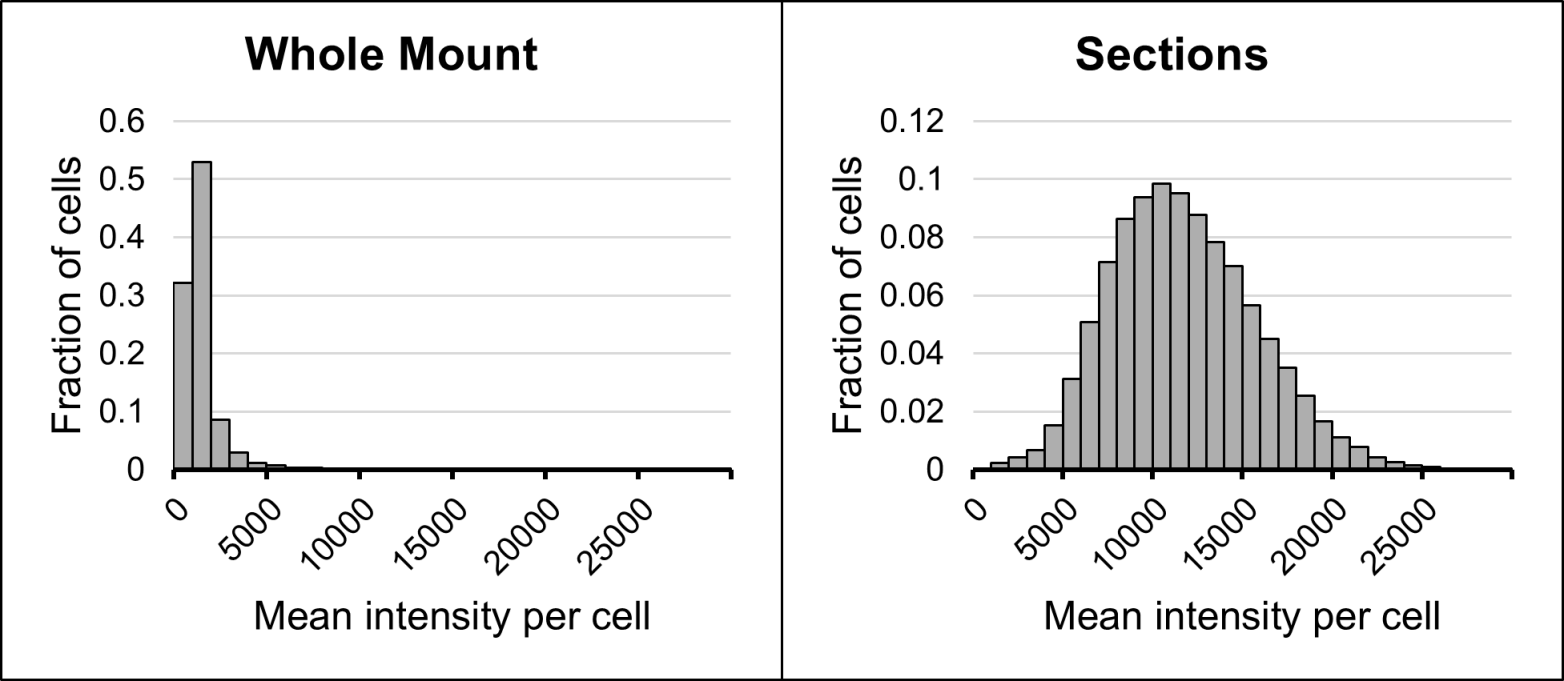
Most cells in sections display a strong signal. Intensity histograms for *S. salivarius* are shown as the fraction of total cells in an intensity bin vs mean intensity per cell in that bin. Bin size: 1,000 arbitrary intensity units. Most cells in whole mount preparations showed an undetectable or weak hybridization signal. In contrast, cells in sections showed increased signal intensity with a normal distribution. Histograms were generated after segmentation of cells and measurement of mean intensity per cell. Total cells measured = 74,169 (whole cell mounts); 99,972 (sections).

The relative efficacy of hybridization was quantified by comparing the fluorescence image for each bacterial population to a reference image obtained with differential interference contrast (DIC) microscopy. The DIC image shows all cells in the field of view, whether they are fluorescent or not. In this way, the fraction of hybridized cells and the relative heterogeneity or homogeneity of hybridization could be assessed. Quantification of signal intensity in *S. salivarius* images (Fig. 2) demonstrated that most cells in whole cell mount preparations showed no or weak hybridization signal. In contrast, the majority of cells in sectioned preparations showed a strong signal with cell intensities following a normal distribution centered about a mean value of approximately 10,000 units. Greater than 80% of the cells in whole mount preparations had between 0% and 20% of the average signal in the sectioned cells, indicating that hybridization was improved by almost an order of magnitude through sectioning.

Hybridization of Gram-negative bacteria as whole cell mounts is generally successful without enzymatic treatment. Such results have been widely reported in the literature and obtained by us for all Gram-negative bacteria we have tested. We include as an example one Gram-negative representative, *Pseudoleptotrichia* sp. HMT-221. As expected, hybridization of *Pseudoleptotrichia* in both whole cell mounts and sections was clear and relatively uniform (Supplemental Information Fig. S1).

Additionally, to confirm that the embedding procedure does not lead to non-specific binding of FISH probes to the section, we performed a control experiment using the problematic Gram-positive bacterium *S. odontolytica* (Supplemental Information Fig. S2). Cells in sections were hybridized under the same stringency conditions with the genus-level probe Act118 and the non-specific probe NON338 (38). Both probes were labeled with the same fluorophore, Atto 633, and the same image acquisition and display settings were used to enable comparison between the probes. The signal for the genus-specific probe was bright and homogeneous, while there was no observed signal for the non-specific probe, demonstrating that the embedment does not cause non-specific binding of FISH probes.

### Hybridization in sections depends on proximity to the cut face

**Fig 3:**
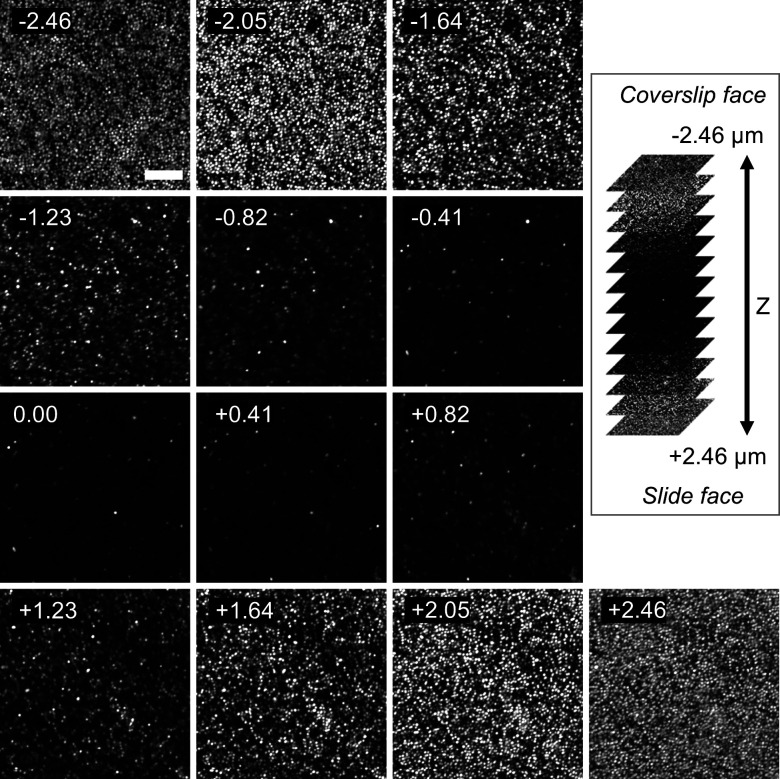
Hybridization in sections depends on proximity to the cut face. Montage of confocal Z-stack throughout a nominally 5 μm thick section of *S. salivarius* hybridized with Eub338-Dy505 probe; 13 optical sections; 0.41 μm optical steps in Z dimension. *S. salivarius* cocci display excellent hybridization at the cut faces in the first 0.8 microns, while cells near the center remain largely undetected. Labels in individual panels denote the Z-position in microns, where zero represents the center of the section. Scale bar = 10 microns. The second image labeled “−2.05” is the same as the image shown in the first row, last column of Fig. 1.

In our sectioning approach, we used a commonly chosen physical section thickness of 5 μm. In contrast, the optical section thickness of our confocal images was below 1 μm, less than 20% of the total physical section thickness. To determine whether the improved hybridization was uniform throughout the volume of the physical section or if the improvement was restricted to the face of the section, we evaluated hybridization throughout the entire section thickness by acquiring images as a function of z-position. A montage of serial optical sections for the Gram-positive bacterium *S. salivarius* (Fig. 3) shows that the hybridization signal was bright and homogeneous at the upper and lower cut faces, but weak and heterogeneous in the interior of the section. The signal strength and heterogeneity of cells in images acquired in the interior 2 μm of the section closely resembled images of the whole cell samples shown in Fig. 1.

**Fig 4:**
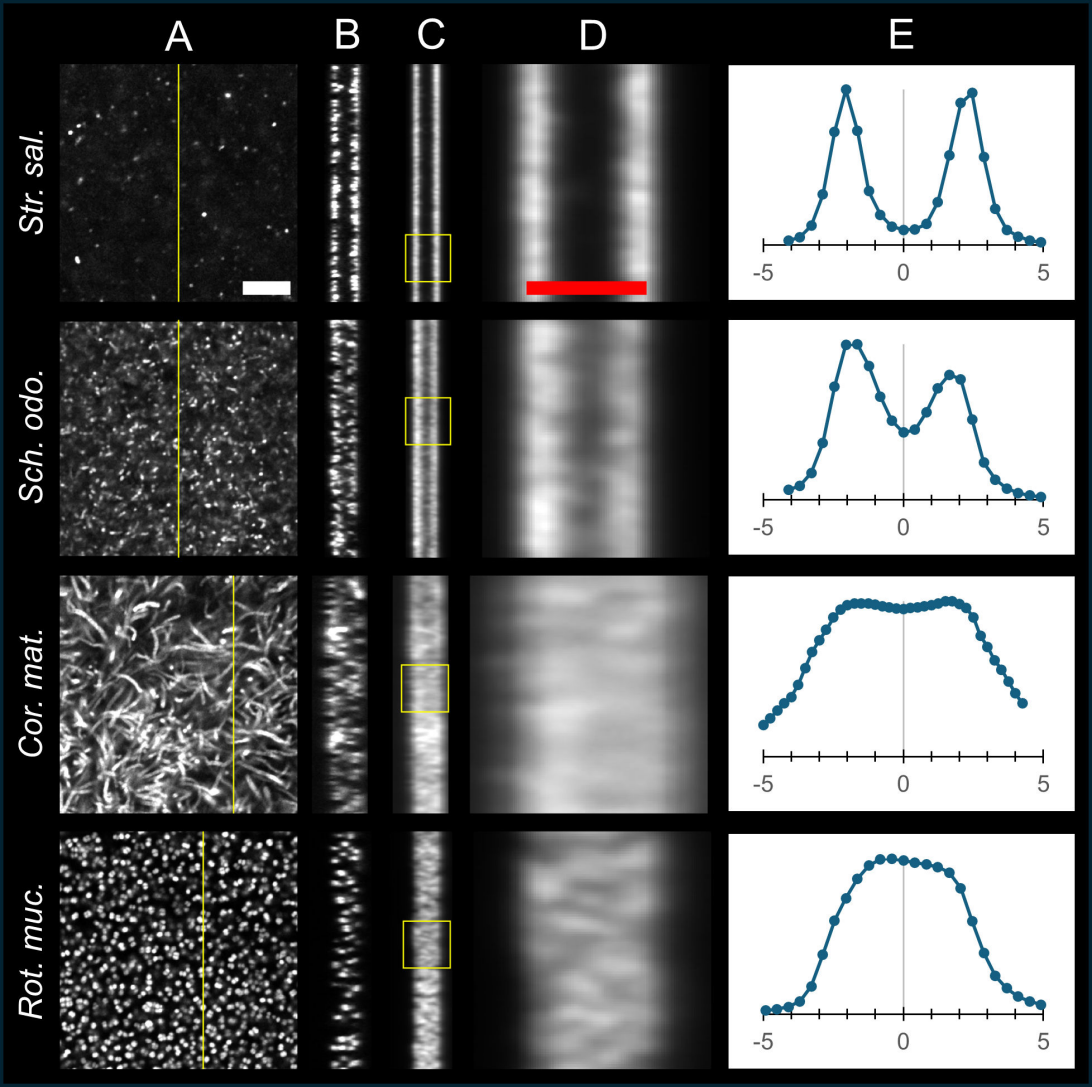
Hybridization in sections depends on bacterial size and morphology. Confocal Z-stacks were acquired from 5 μm thick sections of three difficult-to-hybridize Gram-positive taxa, *Streptococcus salivarius (Str. sal*.), *Schaalia odontolytica (Sch. odo*.), and *Corynebacterium matruchotii (Cor. mat*.), differing in size and shape. A fourth taxon, *Rothia mucilaginosa (Rot. muc*.), that hybridizes readily in whole cell mounts was included as a positive control. (**A**) Fluorescence image of the center focal plane of the physical section. *S. salivarius* and *S. odontolytica* show non-homogeneous hybridization in this plane. (**B**) Orthogonal view of the YZ-plane indicated by the yellow line in (**A**) showing a strong signal at the cut faces and a dramatic reduction of signal in the interior of the section for taxa with cocci or rod morphology, whereas the filamentous taxon, *C. matruchotii*, does not show this reduction. (**C**) Average intensity projection across all YZ planes. (**D**) Enlarged view of the yellow box shown in panel **C**. Hybridization extended further into the section for filamentous *C. matruchotii*. (**E**) Plots displaying average image intensity across all Z-positions in microns, where zero represents the center focal plane of the physical section. The morphology of the bacterium correlates with the level of hybridization in the interior of the section. White scale bar = 10 microns. Red scale bar = 5 microns. The image shown in the first row of column A is the same as the image labeled “0.00” in Fig. 3.

A question posed by these results was whether the weak hybridization in the interior of the section seen for *S. salivarius* was a consequence of poor probe penetration through the methacrylate resin or reflected an intrinsic property of the bacterium in being difficult to hybridize. To address this question, we imaged in the same way the Gram-positive bacterium, *R. mucilaginosa,* which hybridizes readily in whole cell preparations. We observed a uniform hybridization signal at both the cut faces and the interior of the section (Fig. 4, bottom row). The constancy of the *R. mucilaginosa* signal throughout the section depth implies that the FISH probes can penetrate throughout the entire section. This indicates that the embedding resin *per se* does not pose any obstacle to hybridization in the interior. Therefore, the hybridization differences seen for *S. salivarius* at the cut faces versus the interior of the section are related to a property of the bacterium itself.

### Hybridization in sections depends on bacterial size and morphology

We wondered whether the bacterial property responsible for differential hybridization in the interior versus the cut faces of sections was related to bacterial size or morphology. Consequently, we examined other difficult-to-hybridize Gram-positive bacteria differing in size and shape. We collected confocal Z-stacks through sections of the different taxa, obtained the orthogonal YZ view for each X position, and then averaged the intensity projection across all YZ planes (Fig. 4A through D). From the Z-stacks, we also measured the average fluorescence intensity in the XY plane as a function of Z-position (Fig. 4E). In the sections of *S. salivarius,* a small coccoid bacterium of less than 1 μm diameter, we observed a dramatic reduction in hybridization signal within the interior of the section as compared to the face (Fig. 4, top row). Similarly, *S. odontolytica*, which is commonly rod-shaped but can exhibit a range of morphologies, showed a drop in signal intensity, although it was less pronounced (Fig. 4, second row). For *C. matruchotii,* which is pleiomorphic but commonly occurring as long filaments, we observed almost no decrease in hybridization signal throughout the section thickness (Fig. 4, third row). Similar results were obtained for *A. naeslundii* and *C. durum,* which have similar morphology (Supplemental Information Fig. S3). A possible explanation for these results is that bacterial size and morphology contribute to the depth of hybridization. The spherical shape and size of less than 1 μm in diameter of *S. salivarius* correlate well with the observed hybridization depth of approximately 1 μm, as measured by the full width at half maximum of the mean intensity peaks in the Z-intensity profile (Fig. 4E, top). *S. odontolytica* is a slightly larger, pleiomorphic rod bacterium of a maximum length of approximately 2 μm, which corresponds with the increased signal depth observed. *C. matruchotii* is a filamentous bacterium that can grow to more than 30 μm in length (39), a value exceeding the section thickness. These results demonstrate that the morphology of the bacterium, small vs large, coccoid vs filamentous, strongly correlates with the level of hybridization in the interior of the section.

### Hybridization of cells in a complex community

**Fig 5:**
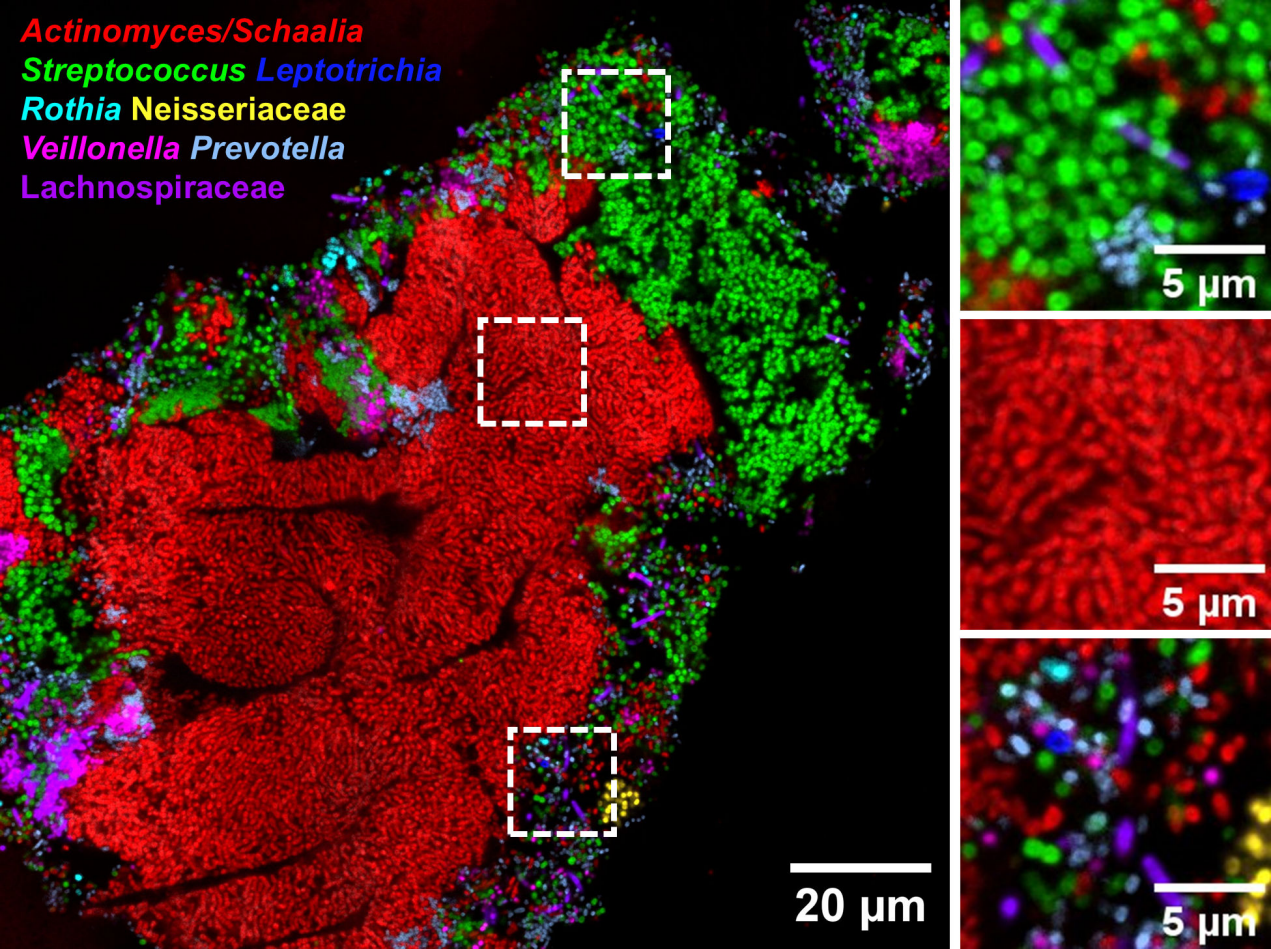
Sectioning enables visualization of complex bacterial communities. Five-micron-thick sections of tongue dorsum biofilm hybridized with probes targeting the problematic Gram-positive bacteria *Actinomyces*, *Schaalia*, and *Streptococcus*, along with six other abundant taxa. *Actinomyces* and *Schaalia* are detected as individual branching rods with a strong signal, while *Streptococcus* cocci exhibit a strong and homogeneous signal. Clusters of intermixed taxa with distinct signals are also observed. Right panels: high magnification images of the dotted squares in the left panel.

The human oral microbiome is the second largest microbial community after the gut. Within it, the tongue dorsum biofilm is particularly dense and spatially organized, consisting of consortia formed by multiple layers of bacteria and extracellular polymeric substances (20). To demonstrate the applicability of sectioning to complex, naturally occurring microbial communities, we hybridized five-micron sections of tongue dorsum biofilm targeting the problematic Gram-positive bacteria *Actinomyces*, *Schaalia*, and *Streptococcus* (Fig. 5), along with six other abundant taxa. *Actinomyces* and *Schaalia* were detected as individual branching rods with a strong signal, while *Streptococcus* exhibited a strong and homogeneous signal with well-differentiated cocci. In addition, diverse taxa were visualized in intermixed clusters with bright and distinct signals, demonstrating the effectiveness of sectioning across a range of bacterial types, even within complex and heterogeneous communities.

## DISCUSSION

**Fig 6:**
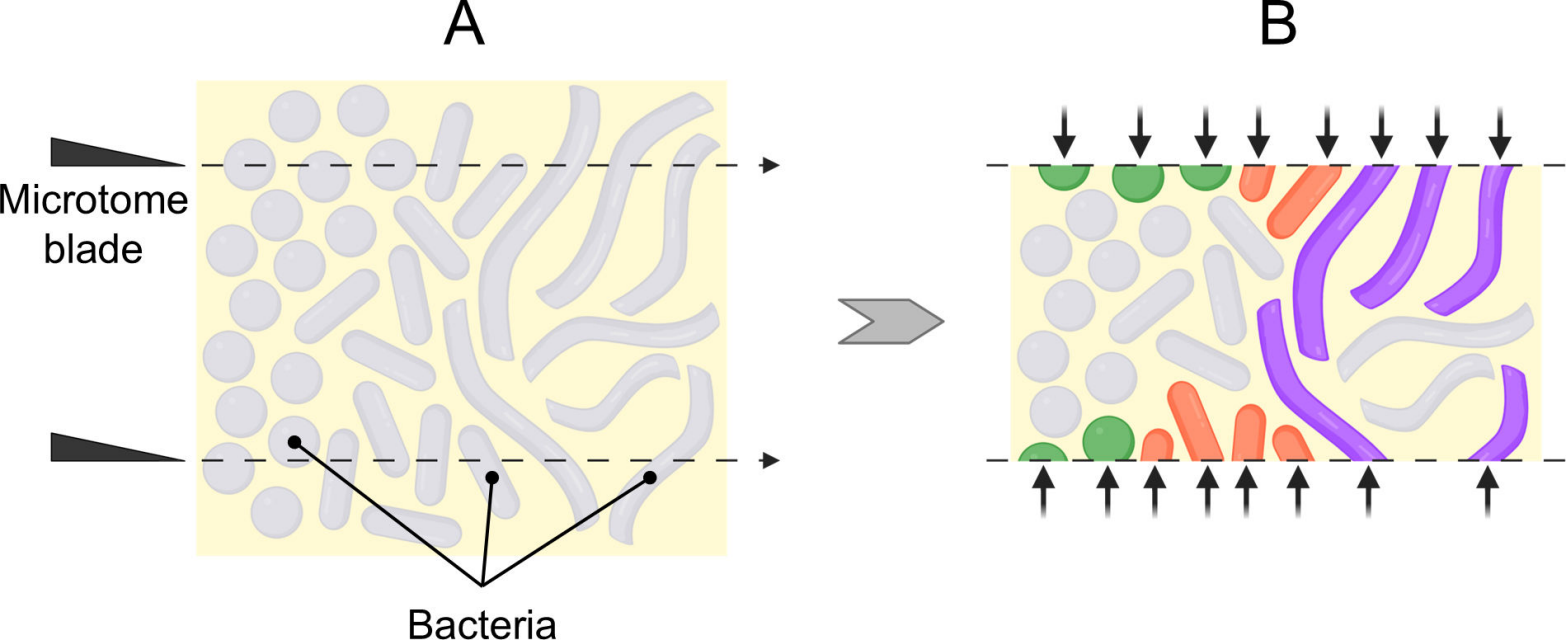
Mechanism of improved hybridization. (**A**) Cocci, bacilli, and filamentous bacteria embedded in a methacrylate resin are sectioned using a microtome blade. The dashed lines represent the slicing path of the blade. (**B**) Fluorescent oligonucleotide probes targeting the specific taxa are applied to the section. Points of access into the cells are indicated by the arrows. Green, orange, or purple colors represent successful hybridization, while gray color represents unhybridized cells. Probes that enter a cell through a cut surface can diffuse and hybridize to target molecules throughout the cell. Created in BioRender. Gundrum, J. (2025) https://biorender.com/ttmcjz8.

Our results demonstrate that the procedure of embedding and sectioning allows the hybridization of both Gram-positive and Gram-negative bacteria without the need for any enzymatic or chemical treatment, thus offering the potential of a universal procedure for effective hybridization. The mechanism by which embedding and sectioning overcome the cell wall barrier is likely simple; namely, that sectioning obviates the barrier by physically slicing through the cell wall (Fig. 6). Probes can then access the interior of the cells and bind to target molecules. This interpretation is consistent with our observation that small coccoid cells, such as *S. salivarius* (~0.8 μm diameter), were effectively hybridized almost exclusively at the faces of sections (Fig. 3). At the section face, the cells will be cut open, but deeper than ~1 μm into the section, no cells would be cut open and hybridization would be restricted by the cell wall barrier as for whole-cell mount preparations. The dependence of hybridization on cell morphology further supports our interpretation of the mechanism. The filamentous bacterium *C. matruchotii* can grow to many microns, even tens of microns in length (39). Such filaments have a greater probability of being cut than small coccoid cells. *Corynebacterium* showed greater hybridization throughout the thickness of the section than *Streptococcus* (Fig. 4). We interpret this to mean that a filament cut at the surface of the section but nevertheless extending deep into the section would be hybridized all along its length because the probe could access the cell interior through the cut opening of the bacterial cell and diffuse throughout the cell interior. The fixed bacterial cytoplasm apparently does not pose a significant barrier to diffusion of the probe throughout the cell.

Since their introduction as embedding media (40), methacrylic resins have become a well-established approach in light microscopy and are fully compatible with a wide range of labeling techniques, including routine histological staining (41), immunohistochemistry (42, 43), and fluorescence *in situ* hybridization. Their hydrophilicity, low contraction during polymerization (44), and excellent morphological preservation further support their broad applicability. Importantly, because factors such as monomer-to-crosslinker ratio and the size of dyes and probes influence penetrability, staining efficiency, and hybridization (45), careful consideration must be given to resin formulation and protocol design.

Our results demonstrate that embedding and sectioning enable effective hybridization of bacteria that are otherwise difficult to probe, while preserving the spatial organization of all taxa within a biofilm. This approach, in combination with fluorescence *in situ* hybridization, provides a robust strategy for visualization of multispecies bacterial communities and is broadly applicable across complex microbial biofilms (17, 25–35). Thus, the integration of embedding and sectioning with FISH not only overcomes longstanding technical barriers but also establishes a versatile platform for advancing the micron-scale spatial organization research of microbiomes in natural contexts.

The sectioning approach also has limitations. Although FISH probes would be able to access large or filamentous bacteria anywhere through the depth of a typical 5 μm section, small coccoid bacteria would be accessible to probes only near the cut face. This means that a complete representation of a complex community, including small bacteria, would be achieved only near the cut face. Alternatively, one could cut thinner sections. Given our results with small coccoid bacteria, we estimate that sections of thickness 1 mm would be sufficiently thin to detect all bacteria. Although thin sections can be more difficult to work with, we have found that, as expected, they give excellent visualization of all taxa probed (36). Another limitation is the time required to carry out embedding and sectioning as compared to whole mount procedures. Embedding and sectioning are low-throughput procedures, necessitating a major commitment of research resources. In contrast to whole mount preparations, it is not well-suited to exploratory research where multiple parameters need to be investigated. A third limitation is the small volume of biological sample that can be examined within a single section. This limits imaging of three-dimensional relationships within the sample. Although multiple serial sections can be cut to reconstruct the sample in three dimensions, the extent of the reconstruction is hindered by technical issues, such as uneven stretching of individual sections, leading to difficulties in registration and computation. Again, three-dimensional reconstruction via serial sections is a low-throughput process as compared with confocal examination of whole mount preparations.

Nevertheless, the rendering of difficult Gram-positive bacteria accessible to FISH by physically slicing them open is an overriding advantage when the biological problem calls for investigating the micron-scale structure of polymicrobial communities containing a mixture of Gram-positive and Gram-negative bacteria. We believe this universal hybridization procedure will be a useful adjunct to other imaging approaches for studying the microbiogeography of microbiomes.

## MATERIALS AND METHODS

### Bacterial pure cultures and fixation

All bacterial strains were cultured in broth consisting of Tryptic Soy and Brain Heart Infusion (Becton Dickinson) supplemented with 10 mg/mL yeast extract and 5 µg/mL hemin and grown at 37°C. *S. salivarius* (ATCC 7073), *R. mucilaginosa* (ATCC 25296), and *C. matruchotii* (ATCC 14266) were grown under aerobic conditions. *S. odontolytica* (ATCC 17929), *A. naeslundii* (F0664)*,* and *C. durum* (F0235) were grown under microaerophilic conditions (2% O_2_ and 5% CO_2_). *Pseudoleptotrichia* sp. HMT-221 (F0705) was grown in an anaerobic chamber (Coy Laboratory Products) with an atmosphere comprised of 5% H_2_, 10% CO_2_, and 85% N_2_. Bacteria were grown to the exponential phase, harvested by centrifugation, washed with phosphate buffered saline, and then fixed in 2% paraformaldehyde in phosphate-buffered saline on ice for 90 minutes. Fixed cells were washed with 10 mM Tris, pH 7.5, and stored in 50% ethanol at −20°C until use. An aliquot of fixed cells was separated for performing FISH on whole cells, while the rest was used for embedding and sectioning.

### Embedding and sectioning

Samples stored in 50% ethanol were dehydrated in acetone for a total of 2 hours on ice, replacing with fresh acetone every 30 minutes. After dehydration, samples were infiltrated with Technovit 8100 glycol methacrylate infiltration solution (Electron Microscopy Sciences) for 2 hours on ice, with the solution being refreshed after 1 hour, followed by overnight infiltration at 4°C. Samples were then transferred to Technovit 8100 polymerization solution and solidified overnight at 4°C in embedding capsules (BEEM embedding capsules, Electron Microscopy Sciences) sealed to exclude air. After solidification, the blocks were desiccated for at least 24 hours. Sectioning was performed using a microtome (ThermoSci Microm HM 355S) with a carbide blade or an ultramicrotome (RCM PowerTome XL) with a glass knife to achieve 5 µm thickness. The resulting sections were collected dry with tweezers and placed directly onto 10 µL drops of distilled water on UltraStick slides (Thermo Scientific). Due to their hydrophilicity, the sections spread evenly on the water, and any folds were gently removed with an eyelash manipulator. Only minimal tearing or section loss was observed. Slides were dried on a 42°C warm plate and stored in dark, dry conditions at room temperature until use.

### Fluorescence *in situ* hybridization

Whole cells and sections were labeled by hybridization with custom fluorescent oligonucleotide probes (Biomers) (Supplemental Information Tables S1, S2 and S3). Hybridization of whole cells was performed in microcentrifuge tubes containing 50 µL of hybridization solution (900 mM NaCl, 20 mM Tris, pH 7.5, 0.01% SDS, 20% [vol/vol] formamide, with each probe at a final concentration of 2 µM) by incubating at 46°C for 2 hours. After hybridization, the buffer was removed, and the cells were washed with 400 µL of washing buffer (215 mM NaCl, 20 mM Tris, pH 7.5, 5 mM EDTA) for 15 minutes at 48°C. Following the wash, the cells were resuspended in 100 µL of washing buffer, and 30 µL were applied to UltraStick slides (Thermo Scientific) and incubated overnight at 4°C to allow the cells to settle and adhere to the slide. After incubation, the slides were rinsed by dipping them into 50 mL of ice-cold water to remove excess salt, followed by dipping into 100% ethanol for quick drying, and then air-dried. Samples were mounted using ProLong Gold antifade reagent (Invitrogen) with a #1.5 coverslip and cured in the dark at room temperature for 24 hours before imaging.

For sections, 50 µL of hybridization solution (900 mM NaCl, 20 mM Tris, pH 7.5, 0.01% SDS, 20% [vol/vol] formamide, with each probe at a final concentration of 2 µM) was applied to each section, followed by incubation at 46°C for 2 hours in a humid chamber. After hybridization, slides were washed by incubation in 50 mL of washing buffer (215 mM NaCl, 20 mM Tris, pH 7.5, 5 mM EDTA) for 15 minutes at 48°C. Sections were rinsed by dipping the slide into 50 mL of ice-cold water to remove excess salt and finally dipped in 100% ethanol for quick drying. Samples were mounted using ProLong Gold antifade reagent (Invitrogen) with a #1.5 coverslip and cured in the dark at room temperature for 24 hours before imaging.

### Image acquisition and linear unmixing

Spectral confocal images were acquired using either an LSM 780 or LSM 980 (Carl Zeiss). In brief, images were acquired with a 32-element spectral detector after excitation with a combination of laser lines 405, 488, 561, 633, or 639 nm, depending on the fluorophores contained in the sample. Objectives used were Plan-Apochromat 63× or 40× 1.4 N.A. with oil immersion. The pinhole was set to 1 AU, and the pixel size was around 0.1 μm to allow resolution of individual cells. Z-series were acquired to capture the entire thickness of the samples using an optimal Z-step size. When needed, a 3 × 3 median filter, followed by weighted linear unmixing, was performed using Zeiss ZEN software to separate fluorophores with close emission spectra. Reference spectra for linear unmixing were acquired from cultured cells hybridized with the Eub338-I probe, single-labeled with fluorophores included in the probe set, and imaged using the same imaging settings as samples. Images were prepared for visual presentation using Fiji software (46).

### Fluorescence intensity analysis of *S. salivarius*

In whole cell mount samples, fields of view containing single layers of cells were imaged as Z-stacks. Fiji software (46) was used for the subsequent analysis. The maximum intensity Z-projection of the fluorescence channel was obtained. Segmentation of bacterial cells was accomplished by applying the “Bandpass Filter” to the brightfield images to even out the background, followed by the “Huang Auto Threshold” algorithm to threshold the cells and then the “Dilate” function. The “Analyze Particles” function was utilized to measure the mean fluorescence intensity from the Z-projection of all segmented cells larger than 0.1 μm^2^.

In the sections, segmentation via brightfield was not possible due to the tightly packed cells. Instead, a maximum intensity Z-projection of the fluorescence image was obtained from the 3 Z-slices at the cut face of the section closest to the slide surface. A 3 × 3 median filter was applied, followed by a low minimum threshold of 1,000. Cell segmentation was achieved by applying the “Find Maxima” function on the filtered image and multiplying this by the thresholded image. The “Analyze Particles” function was used to measure the mean fluorescence intensity from the Z-projection of all segmented cells larger than 0.1 μm^2^.

## Data Availability

All images used for the generation of figures and data analysis were deposited in the public repository Zenodo in their raw Zeiss czi format under the DOI: 10.5281/zenodo.17379566.

## SUPPLEMENTAL MATERIAL

Figure S1 spectrum.02001-25-s0001.TIF Hybridization of the Gram-negative bacterium *Pseudoleptotrichia* sp. is clear and uniform in both whole cell mounts and sections.

Figure S2 spectrum.02001-25-s0002.TIF Embedding bacteria does not cause non-specific retention of FISH probes.

Figure S3 spectrum.02001-25-s0003.TIF Hybridization throughout section depth of three species.

Supplemental material spectrum.02001-25-s0004.docx Supplemental tables and figure legends.

ASM does not own the copyrights to Supplemental Material that may be linked to, or accessed through, an article. The authors have granted ASM a non-exclusive, world-wide license to publish the Supplemental Material files. Please contact the corresponding author directly for reuse.
