# Supplementary figures and images for: Slicing overcomes the bacterial cell wall barrier to fluorescence in situ hybridization
Source: Microbiol Spectr. 2025 Dec 10;14(1):e02001-25. doi: 10.1128/spectrum.02001-25 (PMC12772237; doi:10.1128/spectrum.02001-25)

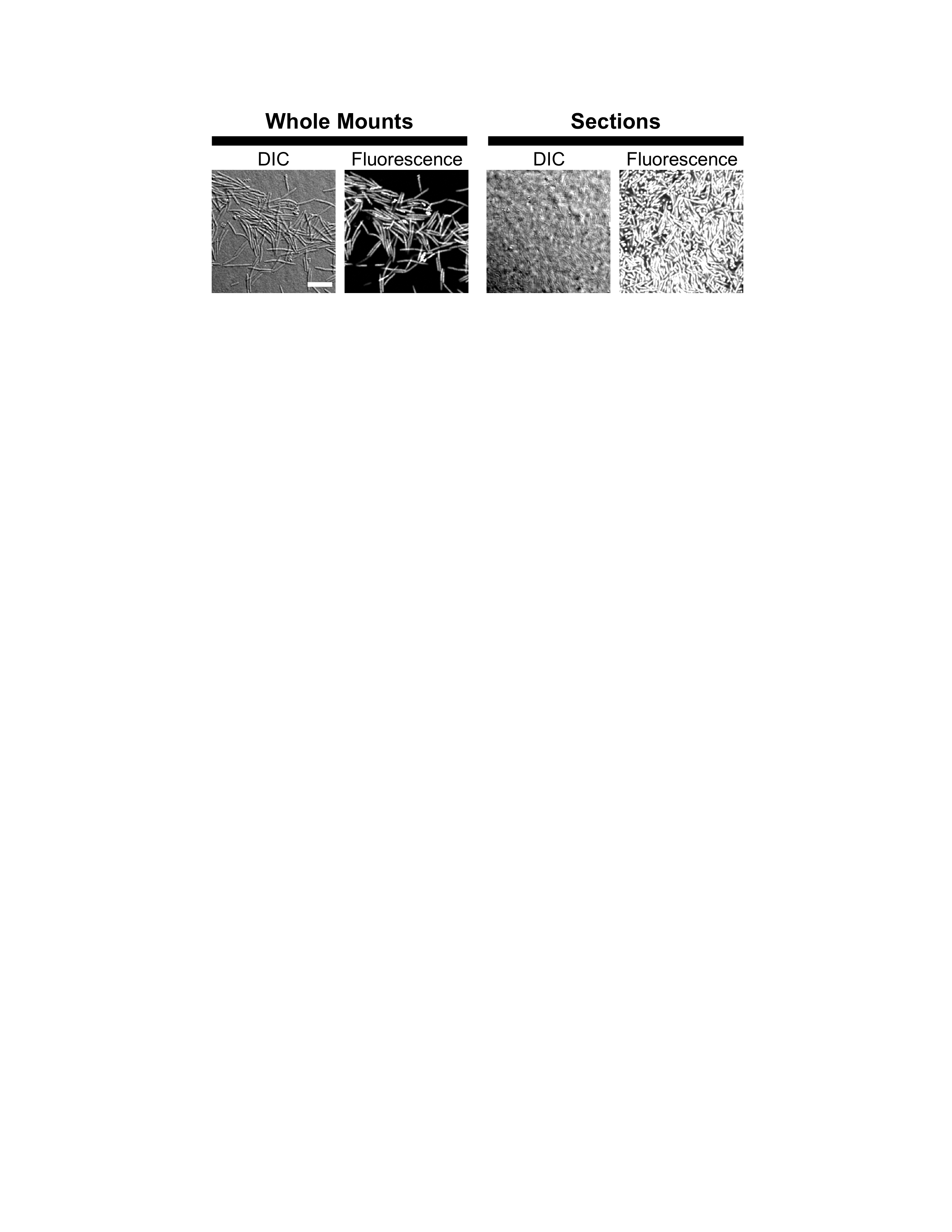

Supplement: Figure S1 — Hybridization of the Gram-negative bacterium Pseudoleptotrichia sp. is clear and uniform in both whole cell mounts and sections. [file spectrum.02001-25-s0001.tif]

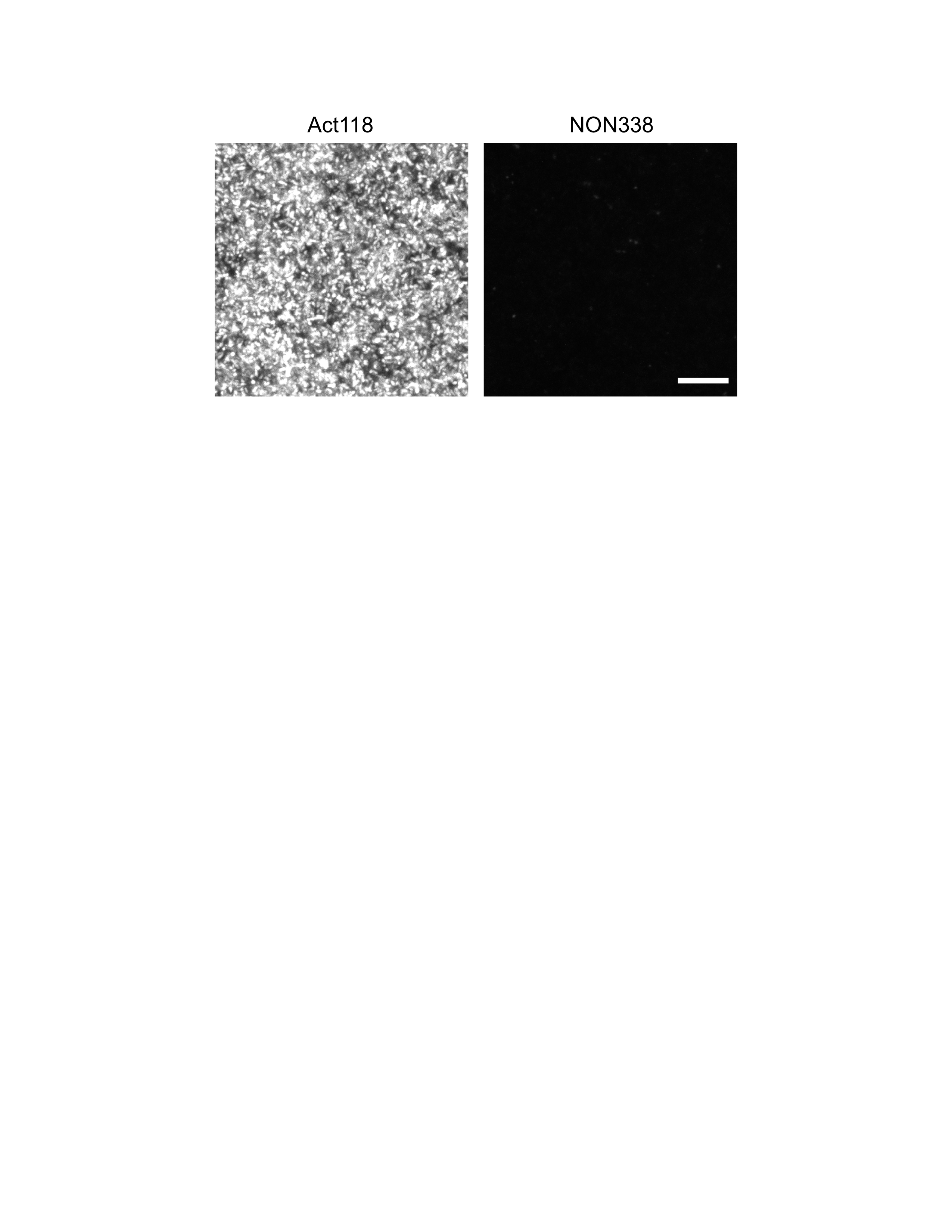

Supplement: Figure S2 — Embedding bacteria does not cause non-specific retention of FISH probes. [file spectrum.02001-25-s0002.tif]

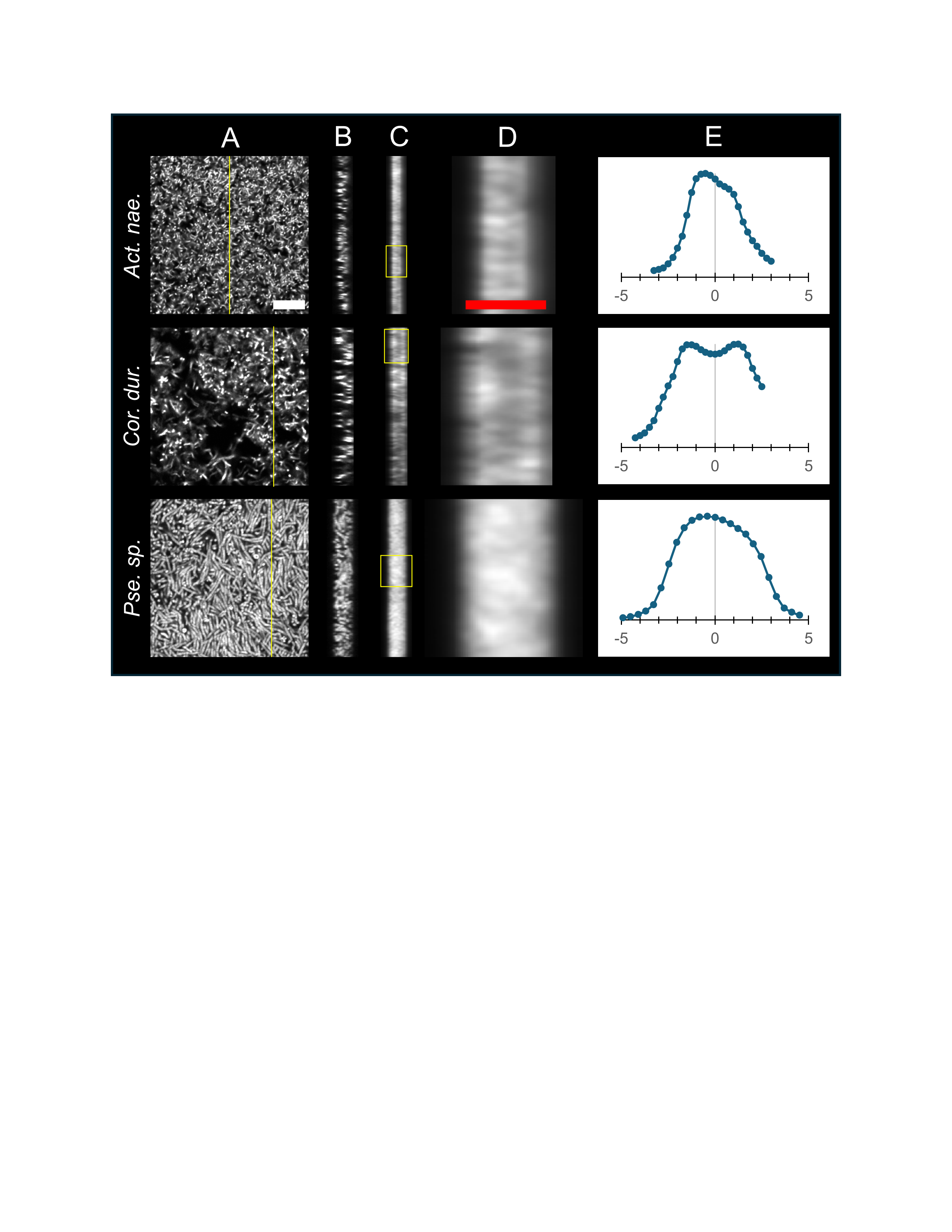

Supplement: Figure S3 — Hybridization throughout section depth of three species. [file spectrum.02001-25-s0003.tif]
