# Supplementary material for: Slicing overcomes the bacterial cell wall barrier to fluorescence in situ hybridization
Source: Microbiol Spectr. 2025 Dec 10;14(1):e02001-25. doi: 10.1128/spectrum.02001-25 (PMC12772237; doi:10.1128/spectrum.02001-25)
Supplement: Supplemental material — Supplemental tables and figure legends. [file spectrum.02001-25-s0004.docx]

**SUPPLEMENTAL INFORMATION**

**Supplemental Table S1. Oligonucleotides used in this study.**

| **Probe Name** | **Target** | **Sequence 5’ – 3’** | **Reference** |
| --- | --- | --- | --- |
| Eub338-I | Eubacteria (Domain) | GCTGCCTCCCGTAGGAGT | (1) |
| Str405 | *Streptococcus* (Genus) | TAGCCGTCCCTTTCTGGT | (2) |
| Ssal1861 | *Streptococcus salivarius* (Species) | ATACCTTCGCTATTGCTAAGC | (3) |
| Act118 | *Actinomyces* (Genus)  *Schaalia* (Genus) | GGCAGGTTACTCACGTGTT | (4) |
| Aodo475 | *Schaalia odontolytica* group (Species) | TTACCCACTACCCTCACCA | (4) |
| Lep568 | *Leptotrichia* (Genus) | GCCTAGATGCCCTTTATG | (5) |
| Rot491 | *Rothia* (Genus) | TAGCCGGCGCTTTCTCTG | (5) |
| Cor633 | *Corynebacterium* (Genus) | AGTTATGCCCGTATCGCCTG | (6) |
| Pre392 | *Prevotella* (Genus) | GCACGCTACTTGGCTGG | (7) |
| Vei488 | *Veillonella* (Genus) | CCGTGGCTTTCTATTCCG | (8) |
| Nei1030 | Neisseriaceae (Family) | CCTGTGTTACGGCTCCCG | (5) |
| Lac432 | Lachnospiraceae (Family) | TCTTCCCTGCTGATAGAGCT | (4) |
| NON338 | Non-specific probe | ACTCCTACGGGAGGCAGC | (9) |

**Supplemental Table S2.** **Probes applied to each strain**. Probe names indicate the oligonucleotide and fluorophore. “Dual” indicates probe was labeled with the same fluorophore at both 5’ and 3’ ends; otherwise, probe was labeled only at the 5’ end.

| **Bacteria Strain** | **Probe Set A** | **Probe Set B** |
| --- | --- | --- |
| *Streptococcus salivarius* ATCC 7073​ | Eub338 - DY 505 Dual | Str405 - Rhodamine Red X Dual  Ssal1861 - Atto 655 Dual ​ |
| *Schaalia odontolytica* ATCC 17929​ | Eub338 - DY 505 Dual ​ | Act118 - Atto 425 Dual ​  Aodo475 - DY 490​ |
| *Pseudoleptotrichia* HMT-221​ | Eub338 - DY 505 Dual  Lep568 - Rhodamine Red X | N/A |
| *Rothia mucilaginosa* ATCC 25296​ | Eub338 - DY 505 Dual ​  Rot491 - Atto 655 Dual ​ | N/A |
| *Actinomyces naeslundii* F0664 | Eub338 - DY 505 Dual  Act118 - Texas Red X Dual | N/A |
| *Corynebacterium durum* F0235 | Eub338 - DY 505 Dual  Cor633 - Atto Rho11 Dual | N/A |
| *Corynebacterium matruchotii* ATCC 14266 | Eub338 - DY 505 Dual  Cor633 - Atto Rho11 Dual | N/A |

**Supplemental Table S3. Probe set used for tongue dorsum biofilm section.** “Dual” indicates the probe was labeled with the same fluorophore at both 5’ and 3’ ends; otherwise, the probe was labeled only at the 5’ end.

| **Probe name** | **Fluorophore** |
| --- | --- |
| Act118 | DY 490 |
| Prev392 | DY 505 Dual |
| Lep568 | Atto 532 Dual |
| Vei488 | Alexa Fluor 555 |
| Nei1030 | Rhodamine Red-X Dual |
| Str405 | Texas Red-X |
| Lac432 | Atto 633 Dual |
| Rot491 | Atto 655 Dual |

**SI Figure S1. Hybridization of the Gram-negative bacterium *Pseudoleptotrichia sp*. is clear and uniform in both whole cell mounts and sections.** Bacterial cells were hybridized under identical conditions either as whole cell mounts (left) or after embedding and sectioning (right) and imaged with confocal microscopy. DIC images are shown to display all cells in the field of view. Fluorescence images display signal from the FISH probe Eub338-Dy505 and represent the optical section containing the highest fluorescence signal. Image acquisition and display settings were kept constant to allow comparison between the two sample preparations. Scale bar = 10 microns.

**SI Figure S2. Embedding bacteria does not cause non-specific retention of FISH probes**. *S. odontolytica* sections were hybridized under identical stringency conditions with either a genus-specific probe (Act118) or a non-specific probe (NON338). Fluorescence images shown here represent the maximum intensity projections from confocal Z-stacks of the sections. Image acquisition and display settings were kept constant to allow comparison between the two probes. Scale bar = 10 microns.

**SI Figure S3. Hybridization throughout section depth of three species.** Confocal Z-stacks were acquired from 5-μm thick sections of *Actinomyces naeslundii*, *Corynebacterium durum*, and *Pseudoleptotrichia sp*. (A) Fluorescence image at the center focal plane of the physical section. (B) Orthogonal view of the YZ-plane indicated by the yellow line in (A). (C) Average intensity projection across all YZ planes. (D) Enlarged view of the yellow box shown in (C). (E) Plots displaying average image intensity across all Z-positions in microns, where zero represents the center focal plane of the physical section. The section of *A. naeslundii* was only ~3 μm thick, which accounts for why we do not observe a drop in signal towards the center. We observed a slight drop in mean intensity in the center of the *C. durum* section; this bacterium usually exhibits a rod-shaped morphology and occasionally longer filaments. Gram-negative filamentous bacterium *Pseudoleptotrichia sp.* exhibits strong fluorescence throughout the section thickness. White scale bar = 10 microns. Red scale bar = 5 microns.

**REFERENCES**

1. Amann RI, Binder BJ, Olson RJ, Chisholm SW, Devereux R, Stahl DA. 1990. Combination of 16S rRNA-targeted oligonucleotide probes with flow cytometry for analyzing mixed microbial populations. Appl Environ Microbiol 56:1919–1925.

2. Paster BJ, Bartoszyk IM, Dewhirst FE. 1998. Identification of oral streptococci using PCR-based, reverse-capture, checkerboard hybridization. Methods Cell Sci 20:223–231.

3. Ramirez-Puebla ST, Mark Welch JL, Borisy GG. 2024. Improved Visualization of Oral Microbial Consortia. J Dent Res 103:1421–1427.

4. Wilbert SA, Mark Welch JL, Borisy GG. 2020. Spatial Ecology of the Human Tongue Dorsum Microbiome. Cell Rep 30:4003-4015.e3.

5. Valm AM, Welch JLM, Rieken CW, Hasegawa Y, Sogin ML, Oldenbourg R, Dewhirst FE, Borisy GG. 2011. Systems-level analysis of microbial community organization through combinatorial labeling and spectral imaging. Proc Natl Acad Sci 108:4152–4157.

6. Mark Welch JL, Rossetti BJ, Rieken CW, Dewhirst FE, Borisy GG. 2016. Biogeography of a human oral microbiome at the micron scale. Proc Natl Acad Sci 113.

7. Diaz PI, Chalmers NI, Rickard AH, Kong C, Milburn CL, Palmer RJ, Kolenbrander PE. 2006. Molecular Characterization of Subject-Specific Oral Microflora during Initial Colonization of Enamel. Appl Environ Microbiol 72:2837–2848.

8. Chalmers NI, Palmer RJ, Cisar JO, Kolenbrander PE. 2008. Characterization of a *Streptococcus* sp.- *Veillonella* sp. Community Micromanipulated from Dental Plaque. J Bacteriol 190:8145–8154.

9. Wallner G, Amann R, Beisker W. 1993. Optimizing fluorescent in situ hybridization with rRNA‐targeted oligonucleotide probes for flow cytometric identification of microorganisms. Cytometry 14:136–143.
